# Editorial: Neurorobotics and strategies for adaptive human-machine interaction, volume II

**DOI:** 10.3389/fnbot.2023.1354389

**Published:** 2024-01-09

**Authors:** Francesca Cordella, Surjo R. Soekadar, Loredana Zollo

**Affiliations:** ^1^Research Unit of Advanced Robotics and Human-Centred Technologies, Università Campus Bio-Medico di Roma, Rome, Italy; ^2^Clinical Neurotechnology Lab, Charité - Universitätsmedizin Berlin, Berlin, Germany

**Keywords:** user intention detection, human-machine interfaces, robot-aided rehabilitation, assistive neurorobotic technologies, human-machine interaction

The world health organization estimated that about 15% of the world population is suffering of some form of disability that influence the quality of life by limiting the execution of various activities of daily living (ALDs) (World Health Organization, 2022). As awareness regarding the potential advantages of assistive and rehabilitation technologies grows, their adoption is on the rise. However, despite this positive trend, several challenges persist that need to be addressed to enhance their usability, acceptance, and integration into clinical practice (Mitchell et al., 2023). These challenges mainly revolve around the inadequate reliability, robustness and usability of human-machine interfaces and to the limited adaptability of the technologies to the user needs and contexts. It remains a challenging endeavor to craft reliable and intuitive human-machine interfaces customized to the user's particular needs and based on their residual capabilities.

Neurorobotics plays a critical role in advancing solutions that can be customized to specific user needs and adapt to diverse contexts. Its goal is to endow machines with the ability to replicate particular aspects of human cognitive functions and behavior.

In this Research Topic, an overview of the state-of-the-art in assistive neurorobotic technologies is provided and the latest results on systems and strategies for monitoring and reconstructing patient state and intention during human-machine interaction are presented.

The different gathered contributions aimed at proposing innovative approaches to solve some of the main limitations of current available solutions. In the work of Kim et al. a solution to overcome some of the main drawbacks of current available body weight support overground walking training systems, such as the cost, the bulkiness and the necessity of a large workspace, is proposed. Several scientific contributions confirmed that self-paced treadmills enable the user to adjust the walking speed mimicking the overground features, also increasing the user attention to gait with positive effects on brain plasticity. The authors outlined that existing systems have characteristics, such as bulkiness, complex installation, high cost, that make them inappropriate for the clinical environment. To spread the clinical use of these devices, the authors propose a cost/space effective system based on a self-paced treadmill and a 2-wire optimized body weight support mechanism. The system is able to mimic overground walking training while guaranteeing the body weight support and a natural pelvic motion. The results obtained by measuring anterior/posterior position, force control, and pelvic motion on eight healthy subjects demonstrated the system efficacy in supporting the body weight during walking providing affordable unloading force.

Another interesting contribution in the field of gait rehabilitation is given by Zorkot et al. that dealt with the problem of foot drop in post-stroke patients. One of the main limitations of most of the current devices is the lack of a system able to identify the gait cycle. It is a useful feature for ankle orthosis since it could aid gait by assisting in the movements of dorsiflexion, plantar flexion and ankle stability. The low-cost ankle orthosis, i.e., the G-Exos, proposed by the authors has been endowed with this skill demonstrating the potentiality of increasing motor capacity in a dropped foot group of volunteers. Both active and passive elements have been used in the design of the G-Exos and force and magneto-inertial sensors have been adopted to identify the gait phases. The sensory information is used by the G-EXOs to promote assistance for ankle dorsiflexion. The validation of the system functionality and usability was performed on 10 volunteers with foot drop. The obtained results, although preliminary, are encouraging.

The problem of achieving natural movements is important also when a rehabilitation system is physically interacting with humans during reaching tasks, as demonstrated in the work of Tang et al.. In this paper, a variable impedance control (vIC) algorithm and a minimum jerk trajectory, which are characteristic of the human upper-limb during the reaching movement, were proposed and tested on a cable-driven rehabilitation robot with 5 healthy subjects. The robot, designed for the rehabilitation of the shoulders and elbows, is characterized by four cables: three cables are used for motion and the fourth one is used for stabilization. The proposed approach was compared with others in literature demonstrating its capacity in improving the tracking accuracy and trajectory smoothness, and in reducing the interaction force at the same time. The obtained results are therefore very promising demonstrating that a control strategy that mimics human-like characteristics could guarantee precise and seamless tracking performance. Furthermore, the proposed vIC could improve the dynamic adaption of the damping on the basis of both the target position and the tracking error. The approach should be further investigated to assess its clinical effectiveness.

Another field of application presented in this Research Topic includes the investigation of user-technology interfaces. Several challenges are still to be solved to guarantee a natural and intuitive interaction between users and machines, as outlined in the work of Carino-Escobar et al.. In this study, the authors explain that the challenge in controlling EEG-based Brain-Computer Interfaces (BCIs) arises from the combination of the low spatial resolution of EEG and the variability of cortical activations during Motor Imagery (MI). To solve this issue, two different feedback timing strategies, i.e., a continuous and a discrete, were compared for controlling a robotic orthosis. The results obtained on 18 participants, in terms of sensitivity and classification accuracy, demonstrated improved performance using continuous feedback timing. Although they represent a preliminary step in improving MI-based systems further analysis should be performed to demonstrate the approach efficacy during neurorehabilitation protocols.

Still with reference to the interfaces for human-machine interaction, the study of Di Vincenzo et al. analyzed the usability of a Natural User Interface (NUI), which emulates the natural sensorimotor embodied interactions of humans with the environment, to control a simulated drone flying in a virtual environment. The NUI, based on an eye-tracker and hand gesture recognition, was compared with a keyboard-based interface in terms of accuracy, easiness, and naturalness in the use. Even though lower performance was achieved with the NUI with respect to the keyboard-based control, the NUI was evaluated more natural and embodied by the 59 involved users. The obtained results have prompted the identification of potential enhancements for the system, encouraging further exploration of system analysis and usability.

This Research Topic will serve as a valuable reference for readers interested in the current state of assistive neurorobotic technologies and human-machine interfaces. The insights provided are expected to contribute significantly to addressing challenges and problems in the medical and teleoperation fields.

## Author contributions

FC: Writing – original draft. SS: Writing – review & editing. LZ: Writing – review & editing.
